# Adherence to Chemoprophylaxis and *Plasmodium falciparum* Anti-Circumsporozoite Seroconversion in a Prospective Cohort Study of Dutch Short-Term Travelers

**DOI:** 10.1371/journal.pone.0056863

**Published:** 2013-02-25

**Authors:** Sanne-Meike Belderok, Anneke van den Hoek, Will Roeffen, Robert Sauerwein, Gerard J. B. Sonder

**Affiliations:** 1 Department of Infectious Diseases, Public Health Service (GGD), Amsterdam, The Netherlands; 2 Department of Internal Medicine, Division of Infectious Diseases, Tropical Medicine and AIDS, Academic Medical Center, Amsterdam, The Netherlands; 3 Department of Medical Microbiology, Radboud University Nijmegen Medical Center, Nijmegen, The Netherlands; 4 National Coordination Centre for Traveller’s Health Advice (LCR), Amsterdam, The Netherlands; The George Washington University Medical Center, United States of America

## Abstract

**Background:**

We conducted a prospective study in a cohort of short-term travelers assessing the incidence rate of anti-circumsporozoite seroconversion, adherence to chemoprophylaxis, symptoms of malaria during travel, and malaria treatment abroad.

**Methods:**

Adults were recruited from the travel clinic of the Public Health Service Amsterdam. They kept a structured daily travel diary and donated blood samples before and after travel. Blood samples were serologically tested for the presence of *Plasmodium falciparum* anti-circumsporozoite antibodies.

**Results:**

Overall, the incidence rate (IR) of anti-circumsporozoite seroconversion was 0.8 per 100 person-months. Of 945 travelers, 620 (66%) visited high-endemic areas and were advised about both chemoprophylaxis and preventive measures against mosquito bites. Most subjects (520/620 = 84%) took at least 75% of recommended prophylaxis during travel. Travel to Africa, use of mefloquine, travel duration of 14–29 days in endemic areas, and concurrent use of DEET (N,N-diethyl-meta-toluamide) were associated with good adherence practices. Four travelers without fever seroconverted, becoming anti-circumsporozoite antibody-positive. All four had been adherent to chemoprophylaxis; two visited Africa, one Suriname, one India. Ten subjects with fever were tested for malaria while abroad and of these, three received treatment. All three were adherent to chemoprophylaxis and tested negative for anti-circumsporozoite antibodies.

**Conclusion:**

Travel to Africa, using mefloquine, travel duration of 14–29 days in endemic areas, and use of DEET were associated with good adherence to chemoprophylaxis. The combination of chemoprophylaxis and other preventive measures were sufficient to protect seroconverting travelers from clinical malaria. Travelers who were treated for malaria abroad did not seroconvert.

## Introduction

Half the world’s population is at risk of malaria [1], [2]. An estimated 216 million malaria cases, and 655,000 deaths, occurred in 2010, mostly among the local population in malaria-endemic regions [2]. Malaria also is a threat to the approximately 80–90 million travelers who visit the 106 endemic countries annually.

Travelers can protect themselves against malaria by using antimalarial chemoprophylaxis and preventive measures against mosquito bites. Recommendations for these preventive measures are based on the anticipated infection rate and drug resistance in *Plasmodium falciparum* [3]–[5], and differ by country.

Risk estimates for malaria infection among travelers from nonendemic countries are usually based on transmission rates in endemic populations and reports of infections in returned tourists. Neither estimate correlates well with the risk for travelers [6], [7].

Studies that estimate incidence based on these reports lack information on how many travelers were protected by chemoprophylaxis and how many were treated for malaria abroad. They are, however, valid to estimate trends. In the Netherlands, the incidence of imported *falciparum* malaria among travelers declined from 10.0/10,000 in the year 2000 to 3.4/10,000 travelers in 2007, whereas the proportion of travelers who did not use chemoprophylaxis rose from 47% to 52% [8]. Since most of the malaria cases occur in travelers who fail to use – or adhere to – the appropriate chemoprophylaxis [5], [9]–[11], adherence to chemoprophylaxis is associated with protection against malaria. Prospective studies among travelers are more valid to estimate risks [12]. Most prospective studies are incomplete in their assessment of adherence to chemoprophylaxis combined with serological testing for *P. falciparum* infection [13]–[20]; in fact there is only one study, from 1991–1992, which examined both adherence to chemoprophylaxis in relation to serological testing.

In order to contribute to a more definitive assessment of risks for travelers, we conducted a comprehensive prospective study as to the incidence rate of *Plasmodium falciparum* anti-circumsporozoite seroconversion, adherence to chemoprophylaxis, symptoms of malaria during travel, and malaria treatment abroad.

## Methods

### Ethics Statement

Study protocol was approved by the Medical Ethics Committee of the Academic Medical Center Amsterdam (MEC 06/016). Participants were included only with informed and written consent.

### Study Population

A prospective study was performed among persons attending the travel clinic of the Public Health Service Amsterdam from October 2006 to October 2007. All persons 18 years and older were eligible if they were planning to travel for 1 to 13 weeks to one or more malaria-endemic countries. Countries were grouped in continents according to the composition of macro geographical (continental) regions described by the United Nations Statistics Division [21]. All participants were seen by a doctor or nurse specialized in travel medicine. Based on Dutch national guidelines for travelers’ health advice [22], participants traveling to low-risk malaria-endemic areas (‘low-endemic areas’) were advised about strict preventive measures against mosquito bites without chemoprophylaxis; participants traveling to intermediate- and/or high-risk malaria-endemic areas (‘high-endemic areas’), were advised about strict preventive measures against mosquito bites and antimalarial chemoprophylaxis. Depending on travel destination and travelers’ characteristics, atovaquone-proguanil, mefloquine, doxycycline, or proguanil were recommended in the Netherlands as chemoprophylaxis against infection with *P. falciparum* malaria [22]. Travelers taking mefloquine were advised to start 3 weeks prior to arrival in high-endemic areas, in case of atovaquone-proguanil one day. Travelers taking proguanil or doxycycline start on the day of arrival. Travelers were advised to continue mefloquine, proguanil, and doxycycline 4 weeks after leaving high-endemic areas, and atovaquone-proguanil for 7 days. Participants received a prescription for the appropriate antimalarial chemoprophylaxis, oral and written information about malaria, the use of chemoprophylaxis, and preventive measures against mosquito bites.

### Survey Methods

A standard questionnaire in Dutch or English was used before departure to collect data on sociodemographics, travel history, and purpose of travel (tourism, work or education, or visiting friends and/or relatives [VFR]). Participants were given a thermometer and were asked to take their temperature if they felt feverish. They were also asked to keep a structured travel diary, recording itinerary, use of antimalarial chemoprophylaxis, use of preventive measures against mosquito bites such as a mosquito net and/or an insect repellent containing N,N-diethyl-meta-toluamide (DEET) and/or sleeping in air conditioned rooms, signs of disease (fever), doctor visits, and (self)treatment. Participants made daily diary entries from the day they arrived at their destination to 2 weeks after their return, to encompass the incubation period of *Plasmodium falciparum* malaria and to register adherence to chemoprophylaxis after return. After travel, a nurse checked the diary in the presence of the participant. Participants were then asked if they had taken the advised chemoprophylaxis prior to travel – if applicable.

### Case Definitions

The number of days spent in malaria-endemic areas was defined as ‘exposure time’. Participants were considered ‘adherent’ to chemoprophylaxis if they took at least 75% of their recommended tablets as prescribed during their stay in high-endemic areas. Adherence before and after travel were separately described. The use of DEET, use of a mosquito net, and sleeping in an air-conditioned room were quantified in percentages by dividing the number of days the preventive measure was used by the number of days spent in a malaria-endemic area; and we dichotomized these variables by using the mean of this proportion in the total study population as the cut-off. Fever was self-reported (feeling feverish or thermometer confirmed fever with a temperature of 38°C or higher) starting 1 week after arriving in a malaria-endemic area.

### Laboratory Methods

Before departure and 2 to 6 weeks after return, participants donated venous blood samples for serology. All blood samples were immediately stored at 6°C. Blood samples for serologic testing were centrifuged (Hettich Rotixa 50 S, see APP/407: program 1, 10 min. 3000 rpm (210 g)) and frozen at −80°C within 24 hours until use. The CSP-ELISA (circumsporozoite protein enzyme-linked immunosorbent assay) was used to test for the presence of *P. falciparum* anti-circumsporozoite antibodies according to standard protocols [23]. The optical density (OD) was read at 450 nm (reference filter 620 nm). Positive-control pooled plasma samples from Tanzanian individuals and negative-control plasma samples from Dutch individuals were included in each plate. The threshold for positivity for anti-circumsporozoite antibodies was calculated as the mean OD of the 7 negative control plasma samples plus 3 standard deviations. If the (OD) in serum samples after travel tested positive and showed a ≥2-fold increase compared to the OD value pretravel, these were defined as anti-circumsporozoite antibody seroconversion. This anti-circumsporozoite antibody seroconversion was considered indicative of exposure to *P. falciparum* during present travel.

### Data Analysis

Data analysis was performed with SPSS version 19.0.0.1 (2010, IBM, Somers, USA). Attack rates (ARs) and incidence rates (IRs) were based on positive anti-circumsporozoite antibody seroconversion indicative for exposure to *P. falciparum.* ARs were calculated by dividing the number of study subjects displaying seroconversion in the CSP-ELISA by the total number of participants at risk. IRs per 100 person-months were calculated by dividing the number of travelers with exposure to *P. falciparum* by our calculation of their exposure time. If a traveler was exposed to *P. falciparum*, we used half of their travel duration in endemic area as their exposure time; for travelers without exposure to *P. falciparum,* we used their total travel duration. Pearson chi-square tests of association were used to compare categorical variables between any two groups. Independent determinants for adherence to chemoprophylaxis and for use of DEET were identified by multiple logistic regression analysis and expressed as odds ratios with 95% confidence intervals. A p-value <0.05 was considered statistically significant.

## Results

### Study Population

A total of 945 travelers to malaria-endemic countries were recruited. Of these, 400 (42%) were male (Table 1). The majority (616, 65%) was under 45 years of age, most (783, 83%) had visited tropical or subtropical countries before, and 879 (93%) were born in a Western country; 815 (86%) traveled for holiday; 71 (8%) traveled for work or education, and 59 (6%) were VFR. More than half (529, 56%) stayed less than 14 days in malaria-endemic areas. The most frequently visited continent was Asia (454, 48%); 285 (30%) traveled to Africa and 206 (22%) to Latin America.

**Table 1:** Characteristics of a prospective cohort of short-term travelers from the Netherlands who visited a malaria-endemic area, October 2006–October 2007.

			Travelers			
	No. travelers		High-endemic area^a^		Low-endemic area^b^	
Total	945		620		325	
Sex						
Male	400	42%	265	43%	135	42%
Female	545	58%	355	57%	190	58%
Age group, years						
18–30	312	33%	207	33%	105	32%
31–45	304	32%	197	32%	107	33%
46–59	223	24%	155	25%	68	21%
>/ = 60	106	11%	61	10%	45	14%
Country of birth						
Western country	879	93%	581	94%	298	92%
Non-Western country	66	7%	39	6%	27	8%
Primary purpose of travel						
Tourism	815	86%	519	84%	296	91%
Visiting friends and/or relatives	59	6%	42	7%	17	5%
Work or education	71	8%	59	10%	12	4%
Previous travel to a tropical/subtropical country						
0	162	17%	91	15%	71	22%
1–6 times	546	58%	357	58%	189	58%
6 times or more	237	25%	172	28%	65	20%
Length of stay in endemic area						
</ = 13 days	529	56%	302	49%	227	70%
14–28 days	333	35%	249	40%	84	26%
>/ = 29 days	83	9%	69	11%	14	4%
Travel destination						
Africa	285	30%	279	45%	6	2%
Asia	454	48%	202	33%	252	78%
Latin America	206	22%	139	22%	67	21%

Of the 945 travelers, 620 (66%) were advised of both chemoprophylaxis and preventive measures against mosquito bites. The other 325 participants traveled to low-endemic areas.

### Adherence to Chemoprophylaxis among Travelers to High-endemic Areas

Of the 620 travelers to high-endemic areas who were prescribed chemoprophylaxis, 449 were prescribed atovaquone-proguanil (48%), 91 proguanil (10%), 70 mefloquine (8%), and 10 (2%) other types of chemoprophylaxis, such as minocyclin, doxycycline, or a combination of chemoprophylaxis.

Of 620 travelers, 520 (84%) took at least 75% of the recommended tablets during their stay in high-endemic areas (Table 2). In multivariable analysis, travelers to Africa were significantly more adherent to chemoprophylaxis than travelers to Asia or Latin America (OR 3.5 (95% CI 1.9–6.5) and 2.7 (95% CI 1.4–5.3) respectively); travelers who spent 14–29 days in endemic areas were significantly more adherent compared to travelers who spent either ≤13 days or ≥29 days in endemic areas (OR 2.2 (95% CI 1.2–3.8) and 2.5 (95% CI 1.1–5.6) respectively); travelers who used DEET as an additional preventive measure in more than 50% of days spent in high-endemic areas were significantly more adherent to chemoprophylaxis compared to those who did not use DEET (OR 2.6 (95% CI 1.4–4.8)); and travelers using mefloquine were significantly more adherent compared to travelers using atovaquone-proguanil or proguanil (OR 5.3 (95% CI 1.2–23.1) and 6.0 (95% CI 1.3–27.5) respectively); Of 620 travelers, 466 (75%) took 100% of the recommended advised tablets during their stay in high-endemic areas, with the same determinants as for 75% adherence.

**Table 2:** Determinants for 75% adherence to malaria chemoprophylaxis during travel among a prospective cohort of 620 travelers from the Netherlands to high-endemic areas, October 2006–October 2007.

	Total		Adherent^a^		OR, Univariable analysis, (95% CI)	p-value	OR, Multivariable analysis^b^, (95% CI)	p-value
Total	620		520	84%				
Sex								
Male	265	43%	222	84%	1.00	0.955		
Female	355	57%	298	84%	1.01 (0.66–1.56)			
Age group, years								
18–30	207	33%	169	82%	1.00	0.577		
31–45	197	32%	164	83%	1.12 (0.67–1.87)			
46–59	155	25%	134	86%	1.44 (0.80–2.56)			
>/ = 60	61	10%	53	87%	1.49 (0.65–3.39)			
Country of birth								
Western country	581	94%	486	84%	1.00	0.563		
Non-Western country	39	6%	34	87%	1.33 (0.51–3.49)			
Primary purpose of travel								
Tourism	519	84%	432	83%	1.00	0.610		
Visiting friends and/or relatives	42	7%	37	88%	1.49 (0.57–3.90)			
Work or education	59	10%	51	86%	1.28 (0.59–2.80)			
Previous travel to a (sub)tropical country								
0	91	15%	77	85%	1.00	0.740		
1–6 times	357	58%	296	83%	0.88 (0.47–1.66)			
6 times or more	172	28%	147	85%	1.07 (0.53–2.17)			
Length of stay in endemic area								
≤13 days	302	49%	237	78%	1.00	**<0.001**	1.00	**0.015**
14–28 days	249	40%	227	91%	2.83 (1.69–4.74)		2.15 (1.21–3.81)	
≥29 days	69	11%	56	81%	1.18 (0.61–2.29)		0.87 (0.40–1.88)	
Travel destination								
Asia	202	33%	153	76%	1.00	**<0.001**	1.00	**<0.001**
Africa	279	45%	259	93%	4.15 (2.38–7.24)		3.53 (1.91–6.50)	
Latin America	139	22%	108	78%	1.12 (0.67–1.86)		1.29 (0.75–2.21)	
Type of chemoprophylaxis								
Atovaquon-proguanil	449	72%	374	83%	1.00	**0.009**	1.00	0.071
Mefloquine	70	11%	68	97%	6.82 (1.64–28.43)		5.28 (1.20–23.13)	
Proguanil	91	15%	68	75%	0.59 (0.35–1.01)		0.89 (0.47–1.67)	
Other	10	2%	10	100%				
Use of DEET, percentage								
No	88	14%	65	74%	1.00	**0.020**	1.00	**0.013**
≤25%	75	12%	60	80%	1.42 (0.68–2.96)		1.47 (0.66–3.27)	
26–50%	103	17%	89	86%	2.25 (1.08–4.70)		2.18 (0.99–4.81)	
51–75%	88	14%	81	92%	4.10 (1.65–10.14)		4.70 (1.82–12.14)	
>75%	266	43%	225	85%	1.94 (1.09–3.47)		2.24 (1.20–4.20)	

b In the multivariable analysis the variable ‘type of chemoprophylaxis’ was included without the category ‘other’ because of 100% compliance, so multivariable analysis was done with 610 travelers.

Table 3 shows the adherence to mefloquine, atovaquone-proguanil and proguanil prior to, during, and after stay in endemic area(s) among travelers who started with prophylaxis. The highest adherence percentage for all categories was found among travelers using mefloquine.

**Table 3:** Adherence to the most-prescribed antimalarial chemoprophylaxis among travelers who started with recommended chemoprophylaxis.

	Advised	Started^a^		Prior to reaching endemic area(s)^b^		While in endemic area(s)^c^		After leaving endemic area(s)^d^	
	N	n_1_	n_1_/N	n_2_	n_2/_n_1_	n_3_	n_3_/n_1_	n_4_	n_4_/n_1_
Mefloquine	70	69	99%	63	91%	68	99%	63	91%
Atovaquone-proguanil	449	396	88%	331	84%	374	94%	300	76%
Proguanil	91	76	84%	NA	NA	68	89%	56	74%

NA, not applicable.

### Antimosquito Preventive Measures

Of 945 travelers, 791 (84%) used DEET, 465 (49%) used a mosquito net, and 544 (58%) slept in an air-conditioned room at least once.

Multivariable analysis showed that travelers born in a non-Western country used significantly less DEET and also slept significantly less often under a mosquito net than travelers born in a Western country. Female travelers used significantly more DEET than male travelers. Older travelers used significantly less DEET or mosquito nets than younger travelers. Spending more days in endemic areas was an independent determinant for using less DEET and sleeping less often in an air-conditioned room. Travelers to high-endemic areas in general used a mosquito net significantly more often and slept less often in an air-conditioned room compared to travelers to low-endemic areas. Travelers to Africa or Latin America used a mosquito net significantly more often and slept less often in an air-conditioned room compared to travelers to Asia.

### Anti-circumsporozoite Antibodies

Of 945 travelers, only 938 serum samples were tested using the CSP-ELISA method because the sample was insufficient to test in 7 cases. Of the 938 samples, 4 (AR 0.4% (95% CI 0.1–1.0)) showed an anti-circumsporozoite antibody seroconversion. All 4 travelers were born in the Netherlands, had been 100% adherent to their chemoprophylaxis during travel, and none reported fever or had visited a doctor (Table 4). Of the 4 travelers, 2 stayed in a high-endemic area up to 14 days and 2 between 14 and 29 days; 2 visited Africa, 1 India and 1 Suriname. The overall IR of anti-circumsporozoite antibodies was 0.8 per 100 person-months (95% CI 0.3–2.0).

**Table 4:** Characteristics and symptoms of subjects with anti-circumsporozoite antibody seroconversion for *P. falciparum*, from a cohort of 945 travelers from the Netherlands who visited malaria-endemic areas, October 2006–October 2007.

	Sex	Age in years	Country of birth	Previous travel to tropical/subtropical country	Destination	Country of destination	Length of stay in endemic area^a^	Purpose of travel	Type of chemoprophylaxis	Adherent	DEET use^b^	Visited doctor	Fever
1	F	51	NL	1–6 times	Asia	India	21	Tourism	P	Y	Y	N	N
2	M	59	NL	6 times or more	Africa	Ethiopia	14	Tourism	M	Y	N	N	N
3	M	25	NL	1–6 times	Africa	Gambia	8	Tourism	AP	Y	Y	N	N
4	M	59	NL	N	Latin America	Surinam	3	Tourism	AP	Y	N	N	N

b DEET (N,N-diethyl-meta-toluamide) use in more than the mean use in the study population.

### Fever

Of the 945 travelers, 74 (8%) reported fever after a median period of 17 days (IQR 12–26) in endemic areas. Of these 74, 67 (91%) used the provided thermometer and recorded fever with a median temperature of 38.7°C (IQR 38.2°C–39.6°C). Of the 74, 24 (32%) had visited low-endemic areas and 50 (68%) high-endemic areas. Of 24 travelers who reported fever in low-endemic areas, 13 (54%) sought medical attention abroad, 2 of whom were tested negative for malaria. Of 50 travelers to high-endemic areas, 17 (34%) consulted a doctor abroad, of whom 8 were tested for malaria and 3 were actually treated for malaria. These last 3 travelers had been fully adherent to chemoprophylaxis during travel and tested negative for anti-circumsporozoite antibodies.

## Discussion

In this prospective study with short-term travelers to malaria-endemic countries, self reported adherence to chemoprophylaxis was good. Best adherence was found among travelers to Africa, travelers using mefloquine as chemoprophylaxis, travelers who spent 14–29 days in endemic areas and travelers who were more adherent to use of DEET. Based on seroconversions for anti-circumsporozoite antibodies, we found an overall attack rate (AR) for *P. falciparum* of 0.4% and an overall incidence rate (IR) of 0.8 per 100 person-months.

Adherence to chemoprophylaxis found in our study is in agreement with self reported treatment adherence found in other studies, which ranged from 70%–89% for atovaquone-proguanil and 72%–95% for mefloquine [14], [15], [24], [25]. Travelers to Africa were more adherent to chemoprophylaxis, which is also consistent with other studies [14], [20], [26]. Even though in our travel consultation we do not communicate differences in risk between different high-endemic continents, it is possible that travelers know that malaria risk in Africa is higher than in other continents and therefore are more cautious. This may also be the reason that travelers to high-endemic areas used a mosquito net more often than in other areas. Further, we found good adherence to DEET to be independently related to good adherence to chemoprophylaxis, suggesting that those travelers who were more adherent to one preventive measure were also more likely to follow other preventive measures. These results are in agreement with previous reports [27], [28]. The high level of adherence to mefloquine in our study suggests that this group of travelers experienced minimal side effects, which is supported by data in the daily diaries. One could argue that travelers who did not tolerate mefloquine during previous travel due to side effects chose to use alternative chemoprophylaxis, such as atovaquone-proguanil or doxycycline. However, previous travel experience was univariably not associated with better adherence to mefloquine, atovaquone-proguanil, or doxycycline.

As in our study, previous studies found longer travel duration to be a determinant of nonadherence [24], [29].

Determinants of nonadherence to chemoprophylaxis found in previous studies were visiting friends and/or relatives (VFR), younger age, extensive travel experience, adventurous travel, and (assumed) adverse reactions [20], [24], [26], [29], [30]. We did not find VFR to be less adherent than other groups of travelers. This is probably because Lobel et al and Ropers et al studied travelers flying back from destinations, whereas VFR in our study (only 7% of the total study population) were recruited in a pretravel clinic and are therefore not representative for VFR in general. The VFR in our study were probably more aware of the risk, therefore attended our pre-travel clinic and were also more likely to follow our recommended chemoprophylaxis. Indeed, imported malaria is mostly seen in VFR who did not seek pre-travel health advice and who never intended to use chemoprophylaxis, or who used chemoprophylaxis inadequately [4], [31], [32].

Furthermore, we did not find a relation between travel experience and adherence, but we did find less adherence to chemoprophylaxis in younger travelers, although not significantly.

Studies have shown that detecting anti-circumsporozoite antibodies can be used in nonimmune travelers using chemoprophylaxis as a measure of *P. falciparum* infection [3], [13], [17], [19], [33], [34]. In our study population, 0.4% had a recent infection with *P. falciparum* sporozoites. All 4 travelers were infected in high-endemic areas, none reported fever or malaria treatment, and all had been adherent to chemoprophylaxis. This suggests that the use of chemoprophylaxis protected these 4 travelers from clinical malaria. There are a few other prospective studies on malaria infection using anti-circumsporozoite antibody tests in nonimmune travelers using chemoprophylaxis [13], [17], [19]. The AR of 0.4% we found was lower than the AR found in 1991–1992 by Cobelens et al (1.3%), but their IR of 1.7 per 100 person-months is comparable to our IR (IR 0.8; 95% CI 0.3–2.0). Nothdurft et al published in 1999 an AR of 4.96% and Knappik et al found in 1999 an AR of 0.95%. Differences in ARs could be due to changes in incidences [8], [35], [36], but also to differences in group characteristics, countries visited, or the use of different ELISA tests. Therefore the studies cannot be compared.

Our study is one of the few comprehensive studies that combined *P. falciparum* anti-circumsporozoite seroconversion, adherence to chemoprophylaxis, and collection of clinical malaria data among travelers. The prospective nature of this study with blood samples pre- and post-travel allowed estimating the AR and IR of exposure to *P. falciparum* malaria. The daily diary entries, which minimized recall bias, provided a good record of the use of chemoprophylaxis and antimosquito preventive measures, fever, and treatment for malaria during travel.

Interpretation of our study results may be influenced by a number of shortcomings. First, the daily diary entries could have served as a reminder for travelers to take their chemoprophylaxis and therefore lead to better adherence to treatment. Second, because of the study design, the end date for travelers to fill in their diaries was 2 weeks after return, so we do not know if travelers using mefloquine or proguanil continued chemoprophylaxis after the 2 weeks. Treatment adherence during stay in endemic areas, however, remains most important in protection against infection with *P. falciparum*. Finally, measurement of malaria exposure by anti-CSP seroconversion has limitations due to the methodology. The sensitivity is limited due to small numbers of inoculated sporozoites by infected mosquitoes and their short life span [37]. Development of a detectable anti-CSP response is dose related and requires multiple inoculations [34], [38]. Preliminary data from controlled human malaria infections in malaria naïve Dutch volunteers [39] show that anti-CSP seroconversion occurs in 30% of the volunteers after a single infection with 5 infected mosquitoes (Sauerwein, unpublished). Therefore, the actual number of travelers exposed to sporozoites may be higher for all endemic areas, and our AR and IR likely represent underestimations. The 3 seronegative travelers treated for malaria may have been exposed and infected.

In conclusion, travel to Africa, using mefloquine, travel duration of 14–29 days in endemic areas, and good adherence to DEET were associated with good adherence to chemoprophylaxis. Adherence to chemoprophylaxis in combination with other preventive measures was good enough to protect the travelers who seroconverted from clinical malaria. None of the travelers to low-endemic areas where chemoprophylaxis is not recommended contracted malaria, so there is no reason to adapt the Dutch national guidelines. Similar prospective studies with larger numbers to specific destinations are needed to make more specific destination- dependent advice about the use of chemoprophylaxis.
