# Correction: Adherence to Chemoprophylaxis and *Plasmodium falciparum* Anti-Circumsporozoite Seroconversion in a Prospective Cohort Study of Dutch Short-Term Travelers

**DOI:** 10.1371/journal.pone.0106147

**Published:** 2014-08-18

**Authors:** The PLOS ONE Staff

The footnotes for each table were removed during the typesetting process of this article. The publisher apologizes for this error. Please see the corrected tables here.

**Table 1:** Characteristics of a prospective cohort of short-term travelers from the Netherlands who visited a malaria-endemic area, October 2006 - October 2007.

			Travelers			
	No. travelers		High-endemic area^a^		Low-endemic area^b^	
Total	945		620		325	
Sex						
Male	400	42%	265	43%	135	42%
Female	545	58%	355	57%	190	58%
Age group, years						
18-30	312	33%	207	33%	105	32%
31-45	304	32%	197	32%	107	33%
46-59	223	24%	155	25%	68	21%
>/ = 60	106	11%	61	10%	45	14%
Country of birth						
Western country	879	93%	581	94%	298	92%
Non-Western country	66	7%	39	6%	27	8%
Primary purpose of travel						
Tourism	815	86%	519	84%	296	91%
Visiting friends and/or relatives	59	6%	42	7%	17	5%
Work or education	71	8%	59	10%	12	4%
Previous travel to a tropical/subtropical country						
0	162	17%	91	15%	71	22%
1 - 6 times	546	58%	357	58%	189	58%
6 times or more	237	25%	172	28%	65	20%
Length of stay in endemic area						
</ = 13 days	529	56%	302	49%	227	70%
14 – 28 days	333	35%	249	40%	84	26%
>/ = 29 days	83	9%	69	11%	14	4%
Travel destination						
Africa	285	30%	279	45%	6	2%
Asia	454	48%	202	33%	252	78%
Latin America	206	22%	139	22%	67	21%

a Travelers to high/intermediate malaria-endemic areas or combined low and high/intermediate areas who were advised about chemoprophylaxis.

b Travelers to low malaria-endemic areas who were advised about preventive measures against mosquito bites.

**Table 2:** Determinants for 75% adherence to malaria chemoprophylaxis during travel among a prospective cohort of 620 travelers from the Netherlands to high-endemic areas, October 2006 - October 2007.

	Total		Adherent^a^		OR, Univariable analysis, (95% CI)	p-value	OR, Multivariable analysis^b^, (95% CI)	p-value
Total	620		520	84%				
Sex								
Male	265	43%	222	84%	1.00	0.955		
Female	355	57%	298	84%	1.01 (0.66 - 1.56)			
Age group, years								
18-30	207	33%	169	82%	1.00	0.577		
31-45	197	32%	164	83%	1.12 (0.67 - 1.87)			
46-59	155	25%	134	86%	1.44 (0.80 - 2.56)			
>/ = 60	61	10%	53	87%	1.49 (0.65 - 3.39)			
Country of birth								
Western country	581	94%	486	84%	1.00	0.563		
Non-Western country	39	6%	34	87%	1.33 (0.51 - 3.49)			
Primary purpose of travel								
Tourism	519	84%	432	83%	1.00	0.610		
Visiting friends and/ or relatives	42	7%	37	88%	1.49 (0.57 - 3.90)			
Work or education	59	10%	51	86%	1.28 (0.59 - 2.80)			
Previous travel to a (sub)tropical country								
0	91	15%	77	85%	1.00	0.740		
1 - 6 times	357	58%	296	83%	0.88 (0.47 - 1.66)			
6 times or more	172	28%	147	85%	1.07 (0.53 - 2.17)			
Length of stay in endemic area								
≤ 13 days	302	49%	237	78%	1.00	<0.001	1.00	0.015
14 – 28 days	249	40%	227	91%	2.83 (1.69 - 4.74)		2.15 (1.21 - 3.81)	
≥ 29 days	69	11%	56	81%	1.18 (0.61 - 2.29)		0.87 (0.40 - 1.88)	
Travel destination								
Asia	202	33%	153	76%	1.00	<0.001	1.00	<0.001
Africa	279	45%	259	93%	4.15 (2.38 - 7.24)		3.53 (1.91- 6.50)	
Latin America	139	22%	108	78%	1.12 (0.67 - 1.86)		1.29 (0.75 - 2.21)	
Type of chemoprophylaxis								
Atovaquon-proguanil	449	72%	374	83%	1.00	0.009	1.00	0.071
Mefloquine	70	11%	68	97%	6.82 (1.64 - 28.43)		5.28 (1.20 - 23.13)	
Proguanil	91	15%	68	75%	0.59 (0.35 - 1.01)		0.89 (0.47 - 1.67)	
Other	10	2%	10	100%				
Use of DEET, percentage								
No	88	14%	65	74%	1.00	0.020	1.00	0.013
≤ 25%	75	12%	60	80%	1.42 (0.68 - 2.96)		1.47 (0.66 - 3.27)	
26 - 50%	103	17%	89	86%	2.25 (1.08 - 4.70)		2.18 (0.99 - 4.81)	
51 - 75%	88	14%	81	92%	4.10 (1.65 - 10.14)		4.70 (1.82 - 12.14)	
> 75%	266	43%	225	85%	1.94 (1.09 - 3.47)		2.24 (1.20 - 4.20)	

a Travelers were considered adherent to treatment if they took 75% of the recommended tablets during stay in high-endemic areas.

b In the multivariable analysis the variable 'type of chemoprophylaxis' was included without the category 'other' because of 100% compliance, so multivariable analysis was done with 610 travelers

**Table 3:** Adherence to the most-prescribed antimalarial chemoprophylaxis among travelers who started with recommended chemoprophylaxis.

	Advised	Started^a^		Prior to reaching endemic area(s)^b^		While in endemic area(s)^c^		After leaving endemic area(s)^d^	
	N	n_1_	n_1_/N	n_2_	n_2/_n_1_	n_3_	n_3_/n_1_	n_4_	n_4_/n_1_
Mefloquine	70	69	99%	63	91%	68	99%	63	91%
Atovaquone-proguanil	449	396	88%	331	84%	374	94%	300	76%
Proguanil	91	76	84%	NA	NA	68	89%	56	74%

a No. of travelers who started with the recommended chemoprophylaxis.

b No. of travelers who took recommended prophylaxis prior to arrival in endemic area(s).

c No. of travelers who took at least 75% of recommended prophylaxis during stay in endemic area(s).

d No. of travelers who took at least 75% of recommended atovaquone-proguanil after leaving endemic area(s) or 75% of recommended mefloquine or proguanil for at least 2 weeks after leaving endemic area(s).

NA, not applicable.

**Table 4:** Characteristics and symptoms of subjects with anti-circumsporozoite antibody seroconversion for *P. falciparum*, from a cohort of 945 travelers from the Netherlands who visited malaria-endemic areas, October 2006-October 2007.

	Sex	Age in years	Country of birth	Previous travel to tropical/subtropical country	Destination	Country of destination	Length of stay in endemic area^a^	Purpose of travel	Type of chemoprophylaxis	Adherent	DEET use^b^	Visited doctor	Fever
1	F	51	NL	1-6 times	Asia	India	21	Tourism	P	Y	Y	N	N
2	M	59	NL	6 times or more	Africa	Ethiopia	14	Tourism	M	Y	N	N	N
3	M	25	NL	1-6 times	Africa	Gambia	8	Tourism	AP	Y	Y	N	N
4	M	59	NL	N	Latin America	Surinam	3	Tourism	AP	Y	N	N	N

AP, atovaquone-proguanil; F, female; M, male; M, mefloquine; NL, the Netherlands; N, no; P, proguanil; Y, yes.

a In days.

b DEET (N,N-diethyl-meta-toluamide) use in more than the mean use in the study population.
